# Interfacial Li^+^ Diffusion Booster Accelerated by Enhanced Metal‐Organic Framework Sieving and Wettability for High‐Voltage Solid‐State Lithium Metal Batteries

**DOI:** 10.1002/cssc.202501351

**Published:** 2025-09-02

**Authors:** Tianhua Chen, Yongzheng Zhang, Simeng Wang, Jin Li, Hongzhen Lin, Dusan Losic, Shimou Chen, Jian Wang

**Affiliations:** ^1^ School of Chemical Engineering and Advanced Materials The University of Adelaide Adelaide SA 5005 Australia; ^2^ Institute of Process Engineering Chinese Academy of Sciences Beijing 100190 China; ^3^ State Key Laboratory of Chemical Engineering East China University of Science and Technology Shanghai 200237 China; ^4^ i‐Lab & CAS Key Laboratory of Nanophotonic Materials and Devices Suzhou Institute of Nano‐Tech and Nano‐Bionics Chinese Academy of Sciences Suzhou 215123 China; ^5^ State Key Laboratory of Chemical Resource Engineering Beijing University of Chemical Technology Beijing 100029 China; ^6^ Helmholtz Institute Ulm (HIU) D89081 Ulm Germany; ^7^ Karlsruhe Institute of Technology (KIT) D76021 Karlsruhe Germany

**Keywords:** interfacial diffusion enhancement, ionic liquid wettability, Li metal anode, sieving effect, solid‐state battery

## Abstract

Solid‐state lithium metal batteries (SSLMBs) are promising for realizing higher energy density. However, the poor interfacial Li^+^ transport kinetics and Li dendrite growth inhibit SSLMBs, leading to sluggish interfacial ion diffusion and depressive lifespan, which is attributed to high barriers blocked by anions or interface space in solid‐state electrolytes. Herein, a flexible solid‐state polymer skeleton employed with ionic liquid and metal‐organic frameworks (PIM) electrolyte is proposed to strengthen interfacial Li ion exchange by improving the Li^+^ sieving effect and interfacial wettability. Thanks to the immobilization effect of TFSI^−^ anions affected by positive metal atom centers and pore morphology, the PIM electrolyte exhibits exceptional properties, i.e., a high ionic conductivity up to 3.1 mS cm^−1^ at 60 °C and an improved Li^+^ transference number of 0.65, enabling symmetric cells of Li metal to run steadily for over 1000 h with lower voltage hysteresis (25 mV). Meanwhile, matching with high‐voltage electrodes, the solid‐state PIM electrolyte exhibits good compatibility and stability toward LiNi_0.6_Co_0.2_Mn_0.2_O_2_ and LiFePO_4_ electrodes, showing the capacity retentions of 85.5% and 96.5% after 120 and 400 cycles, respectively. This work suggests low interfacial diffusion resistances and high compatibility for make it a promising candidate for future solid‐state battery.

## Graphical abstract

An ion‐selective SSE is proposed with an accelerated Li+ migration feature, where the metal organic framework functions as a molecular sieve to selectively block bulky anions and the ionic liquid acts as Li ion bridge. Consequently, The as‐prepared full cell exhibited high capacity and retention under the upper voltage of 4.3 V.

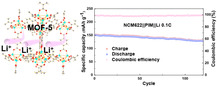

## 1 Introduction

Lithium metal batteries are considered the next most promising batteries for reaching ever‐increasing demands in energy density and safety.^[^ ^1^ ^]^ Compared with liquid carbonate or ether electrolyte, the solid‐state electrolytes (SSEs) show the superiorities in safety, extended working window and enhanced electrochemical stability, attracting the researchers’ pursuit for higher energy density.^[^ ^2^ ^]^ Different from the inorganic sulfide‐based or garnet‐based SSEs,^[^ ^3^ ^]^ the solid‐state polymer electrolytes, i.e., poly(ethylene oxide) (PEO), have been extensively studied due to its compatibility and robustness with various lithium salts and metallic Li surface.^[^ ^4^ ^]^ Despite their potential benefits, all‐solid‐state polymer electrolyte/lithium salt complexes are often constrained by their low ionic conductivity, low Li^+^ transference number, huge interfacial resistance, and potential formation of Li dendrites, thereby limiting their applicability in high‐rate solid‐state lithium batteries.^[^ ^3^, ^5^ ^]^ Meanwhile, matched with high‐voltage cathode, the coupled full cells always lose and encounter the instability, deteriorating the cathode/electrode interface under higher oxidation voltage.^[^ ^6^ ^]^

To overcome these drawbacks, numerous efforts have been dedicated to enhancing the ionic conductivity and Li ion transference of polymer‐based solid electrolytes. Investigations have indicated that nanosized particles endowed with Lewis acidic surface properties or functional groups decorated on the polymer skeleton hold significant promise in enhancing the ionic conductivity of electrolytes.^[^ ^7^ ^]^ However, researches related to PEO‐based SSEs still have insufficient contact with metallic Li anode, especially under high upper voltage, which would shorten the lifespan and broaden the overpotentials with low Li ion transfer kinetics.^[4c]^ To address the issue of interfacial mobility, ionic liquids (IL) are introduced into the solid polymer electrolyte, aiming at enhancing the wettability and ionic conductivity.^[^ ^8^ ^]^ Actually, in the mixed IL‐decorated polymer electrolyte, there are two dual ion promotors, and the ionic conductivity is governed by the free ions and anions. This would result in a gradual concentration of Li ion distribution and cause uneven dendrite formation. Therefore, it is urgent to confine the anion species to uniformize ion diffusion so that to improve the lithium‐ion conductivity.

Alternatively, metal organic frameworks (MOFs), as a porous material family, exhibit the merits in adjustable pore sizes, abundant polar coordination ligands, desirable high conductivity, excellent chemical and thermal stability.^[^ ^9^ ^]^ Recently, it is revealed that introducing MOF into the polymer solutions could form a new type SSE and possess several advantages in providing a unique pathway for the transport of Li^+^ by confining the larger anions, forming the single‐ion conductor.^[^ ^10^ ^]^ For example, by integrating the metal sites and transmission channels, Long et al. produced the first MOF‐based SSE with high ionic conductivity (3.1 × 10^−4^ S cm^−1^).^[10a]^ Additionally, decreasing the MOF particles into nanosize, the surface tension and area are significantly enhanced, and then the contact between the SSE and electrode interface is more intimate, promoting ion transport kinetics.^[^ ^11^ ^]^ In combination with the ILs, Wang et al. prepared a new type of solid‐state electrolyte with the introduction of nanoporous UIO‐66, achieving fast Li^+^ transportation in the solid‐state lithium batteries.^[^ ^12^ ^]^ All these works have demonstrated the enhanced ionic transportation kinetics at the interfaces makes MOF‐derived nanostructured electrolyte more promising candidate in solid‐state batteries. However, issues and the properties of MOF‐derived nanostructure electrolyte in solid‐state batteries such as electrochemical stabilities, electrodes and electrolyte interfacial contact, and their ability to maintain stable Li deposition have not been adequately studied.

In this work, the IL decorated MOF‐5 porous particles are incorporated into a PEO/lithium bis(trifluoromethylsulfonyl)imide (LiTFSI) matrix (named as PIM), forming a solid‐state electrolyte with the capability of high Li^+^ diffusion. In this design, the IL is capable of wetting the Li surface with the decreased contact angle to 89°, shrinking the interfacial resistance. The MOF serves as the sieving mesh to filter the large anion species for averaging ionic transport of Li^+^, generating specific pathway for Li ion transport.^[^ ^13^ ^]^ Benefiting from above merits, the optimized electrolyte results in 10‐times higher improvement of conductivity (3.1 × 10^−3^ S cm^−1^) than pristine PEO electrolyte under 60 °C. Meanwhile, the movement of TFSI^−^ anion has been confined in the porous MOF‐5 structure, highly improving Li^+^ transference number above 0.65 and preventing dendrite formation on Li anodes for stabilizing the lifespan of 1000 h. Consequently, the coupled full cells composed of LiNi_0.6_Co_0.2_Mn_0.2_O_2_ or LiFePO_4_ electrodes deliver the initial specific capacities of 142 mAh g^−1^ at 0.5C and 152 mAh g^−1^ at 0.1C, maintaining the capacity retention of 96.5% after 400 cycles and 85.5% after 120 cycles under high upper voltage, respectively. This study suggests that the interfacial Li^+^ diffusion‐enhanced anion‐immobilized SSEs have great potentials to develop high‐performance lithium metal batteries.

## 2 Results and Discussion

### 2.1 Characterizations of Ion Diffusion‐Boosted Solid‐State Electrolyte

**Figure 1:**
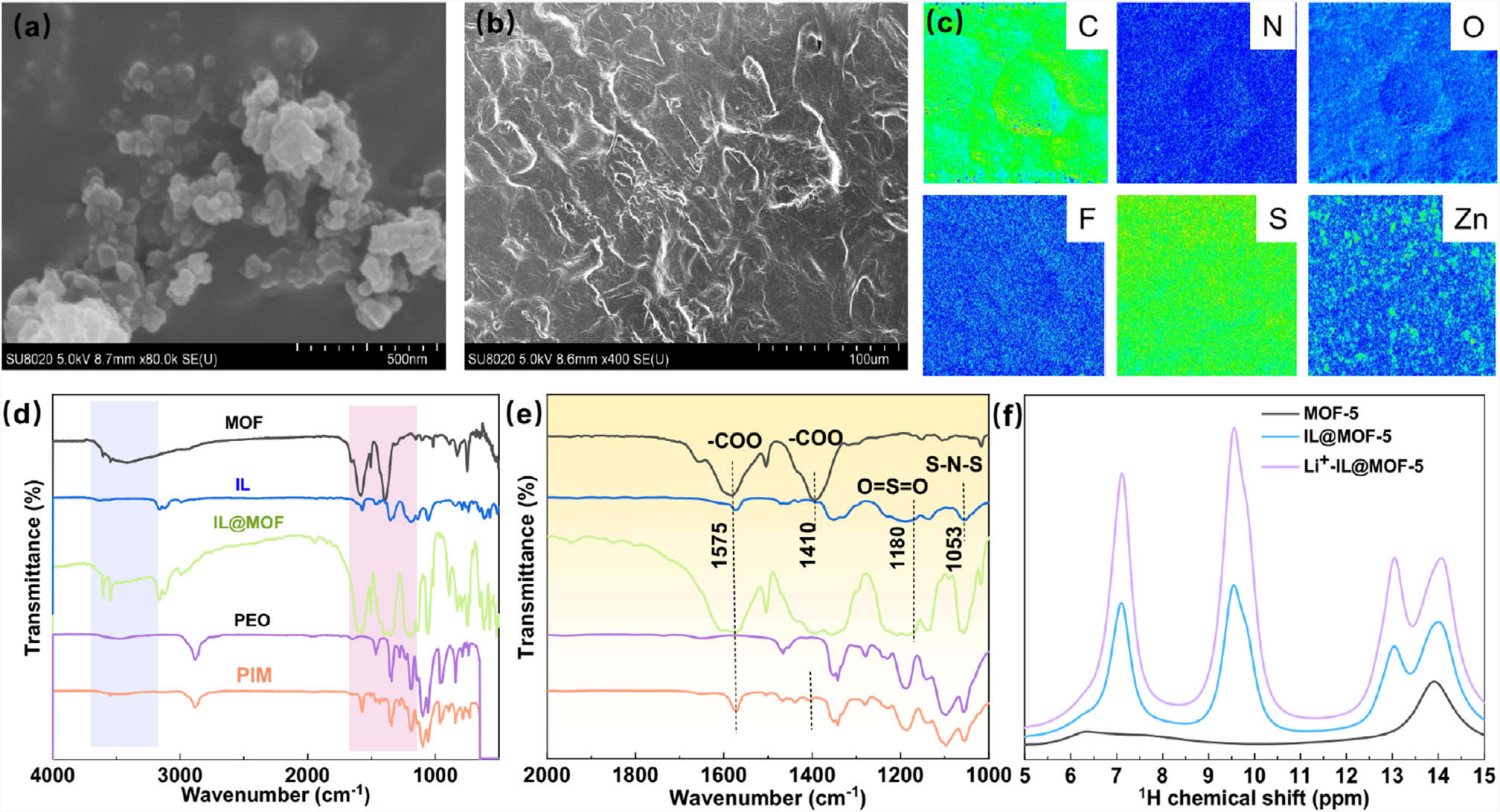
SEM images of a) MOF‐5 and b) PIM film; c) EMPA images of corresponding C, N, O, F, S, and Zn elements in PIM film; d,e) FTIR spectra comparison of MOF, IL, IL@MOF, PEO, and PIM; f) ^1^H NMR spectrum of IL@MOF‐5.

The processing methods of PEO and PIM membrane electrolyte was illustrated in the Figure S1a, Supporting Information, demonstrating the effective dispersion of IL and MOF‐5 nanoparticles in the PEO solution and the feasibility to make the thin membrane electrolyte. In this design, the MOF help to sieve and attract the large anions and the IL help to bridge the ion transport together with the uniform formation of SEI. Specifically, the porous MOF functions as a molecular sieve to selectively block bulky anions of TFSI^−^ through the electrostatic effect between metal center and anions, which is beneficial for homogenizing Li^+^ ion migration. At the same time, the introduction of MOF in the system will also decrease the crystalline of PEO, which is further beneficial for ion transport. In consistency with Figure S1b, Supporting Information, of obvious X‐ray diffraction (XRD) pattern, the MOF‐5 filler can be recognized, and **Figure** **1**a shows that the average particle size of MOF‐5 crystals is around 20–30 nm in the PIM bulk electrolyte.^[^ ^14^ ^]^ Figure S1c, Supporting Information, also evaluated the crystallization of the polymer electrolyte, and the results indicate that the participation of MOF‐5 declines the PEO crystallization temperature from 60 °C to 50 °C in the DSC curves, indicating the feasibility for fast Li ion transport.^[^ ^15^ ^]^

To achieve a functional solid‐state membrane, the gradient and ratios of polymer and Li salts were initially optimized. Figure S2, Supporting Information, shows the optical and scanning electronic microscopy (SEM) images of the different membrane electrolytes with different amount of Li^+^ salt. When the EO/Li^+^ ratio was set to be 16:1, the high‐resolution SEM image shows that the optimal membrane electrolyte exhibited the uniform and relatively flat surface with MOF‐5 nanoparticles well‐distributed throughout the membrane electrolyte (Figure 1b and S2, Supporting Information). Decreasing or increasing the EO/Li^+^ ratios, the surface of PIM is either porous or aggregative (Figure S2, Supporting Information). It can be observed that the addition of MOF‐5 and IL@MOF‐5 nano‐wetted particles significantly decrease the crystallinity and porosity of PEO.^[^ ^16^ ^]^ Among them, the distribution of nanoparticles in PIM was more uniform, which can be attributed to the addition of IL that reduces the electrostatic interactions between MOF‐5 particles, thus improving their dispersibility. Furthermore, the introduction of ILs exerts a diluting effect on PEO, thereby reducing the intermolecular forces between polymer chains, diminishing the proclivity for polymer crystallization, and ultimately decreasing the degree of crystallinity, which is consistent with DSC results. It is widely accepted that a decrease in the degree of crystallinity is beneficial for enhancing the ionic conductivity of polymer matrices. This can be attributed to the promotion of chain segment mobility and increased interfacial contact, which facilitates the migration of lithium ions. As displayed in Figure 1c, the EMPA analysis exhibits the various elements in PIM are uniformly distributed, suggesting that the MOF is well anchored in the membrane electrolyte. Furthermore, the SEM elemental distribution spectroscopy (EDS) mapping images in Figure S3, Supporting Information, can also confirm this well‐distribution of each component such as IL and MOF.

The nanoconfinement effect of MOF‐5 and IL@MOF‐5 in PIM is characterized by Fourier transform infrared (FTIR) spectra, as shown in Figure 1d,e. There are three distinct vibrational regions in the MOF‐5 and IL@MOF spectra,^[^ ^17^ ^]^ and the first major vibrational region ranges from 1390 cm^−1^ to 1690 cm^−1^, where four clear peaks can be observed. In comparison with standard spectra, the curve fluctuations in this region are mainly caused by the *O*—C=O vibrations in the carboxyl functional group of the PEO skeleton. The more prominent peaks at 1585 and 1656 cm^−1^ are caused by the asymmetric stretching vibration of the carboxyl *O*—C=O bond, while the peak at 1393 cm^−1^ shows the symmetric stretching motion of the *O*—C=O bond. The second distinct vibrational region includes adsorption peaks at 748 and 825 cm^−1^, assigned to different C—H bonds in the benzene ring, indicating the successful introduction of the MOF‐5 framework in the PIM electrolyte. Meanwhile, the peaks corresponded to the Zn—O bond are ascribed to the formation of the Zn_4_O tetrahedral metal cluster. However, the peak of ‐OH disappears in the PIM due to the interaction between Li^+^ and the ‐OH group. At the same time, these differences can also be reflected in the range of 1510˜1610 cm^−1^, that is, the fact of Li^+^ coordination with the stretching vibration of carboxyl groups. The characteristic peaks of the MOF become more pronounced after the addition of the IL. Peaks at 1053 and 1180 cm^−1^, which corresponds to the vibrations of S–N–S and O=S=O of the TFSI^−^, respectively, were observed in the IL. Upon the incorporating of the IL in the MOF‐5 framework, the peaks experienced a slight blue shift, implying robust interactions between the TFSI^−^ and the MOF, resulting in an enhanced capture ability toward TFSI anion.^[^ ^18^ ^]^ The FTIR of PIM shows that Li^+^ coordinated with carboxyl groups, enhancing the potential of the adsorption site, and improving the interaction between the site and Li^+^, thereby improving the transport of Li‐ions. In the Figure 1f, compared to MOF‐5, the increased area in hydrogen bonds observed in the ^1^H NMR spectrum of IL@MOF‐5 indicates that the IL is effectively integrated into the MOF‐5 structure. This integration enhances the formation of hydrogen bonds with polyethylene oxide (PEO), leading to a reduction in the crystallinity of PEO, as demonstrated in Figure S2, Supporting Information.

### 2.2 Fast Li Ion Kinetics Achieved by Interface Wettability and MOF Sieving

**Figure 2:**
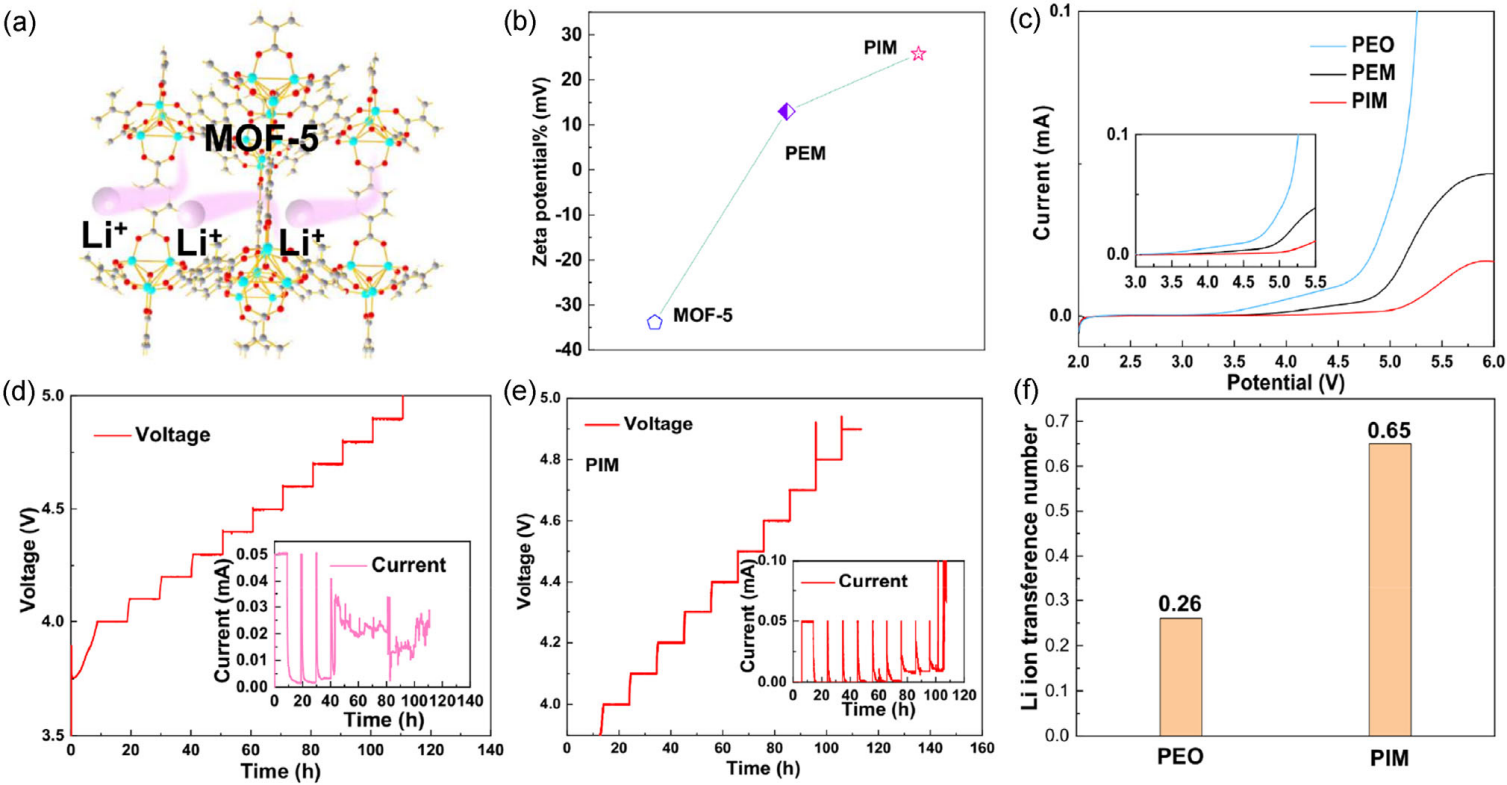
a) Schematic illustration of Li^+^ transportation pathways in the nanowetted IL@MOF‐5 particles; b) Zeta potentials of MOF‐5, PEM, and PIM in acetonitrile suspension; c) LSV curve of Li||PEO||SS, Li||PEM||SS, Li||PIM||SS at 60 °C. Electrochemical floating analysis of d) NCM622||PEO||Li battery; e) NCM622||PIM||Li battery; and f) Li^+^ ion transference number in PEO and PIM electrolytes, respectively.

The lithium‐ion transport kinetics in the solid‐state electrolyte are initially evaluated. As illustrated in **Figure** **2**a, the larger TFSI anion is expected to be immobilized and the lithium ions involves their movement through its pores and transfer between the crystals via framework interconnectivity. In Figure 2b, the zeta potentials of MOF‐5, PEO‐MOF‐5, and PEO‐IL@MOF‐5 were measured and compared in acetonitrile solution, which could be served as a metric for the intensity of inter‐particle repulsion or attraction. The observed change of zeta potentials from ‐33.89 to 25.77 mV signifies a shift from negative to positive, implying that IL@MOF‐5 particles exhibit a robust capability in propelling dissociation for fast lithium ions kinetics. Furthermore, the activation energy of Li ion was assessed by the electrochemical impedance spectroscopy (EIS) measurements under the temperatures ranging from 0 °C to 80 °C (Figure S4a, Supporting Information).^[^ ^19^ ^]^ Remarkably, high ionic conductivities of 3.7 × 10^−5^, 3.3 × 10^−4^, 3.1 × 10^−3^, and 2.8 × 10^−2^ S cm^−1^ were achieved at 0, 25 (room temperature), 60, and 80 °C (Figure S4b, Supporting Information), respectively, suggesting the temperature robustness for PIM membrane electrolyte. Generally, the lower the activation energy, the faster migration of Li ions. According to the classic Arrhenius equation of $\text{σ }\left(T\right)=A\text{exp}(-\frac{\text{Ea}}{RT}$),^[^ ^20^ ^]^ the activation energy barrier of Li ion transport was estimated to be about 0.30 eV, which is only one fifth of that of PEO‐based electrolytes (about 1.66 eV).^[^ ^21^ ^]^

The electrochemical windows of the SSEs were determined by linear sweep voltammetry (LSV) with the asymmetric cell of Li||PIM||SS under 60 °C (Figure 2c). The optimized PIM electrolyte demonstrated a stable electrochemical window between 2 and 5.0 V, while the PEM or PEO will be oxidated under the high voltage of 4.5 V or 4.2 V, respectively. This extended voltage window can be attributed to the stability of the MOF‐5 against oxidation over higher upper potential, which allows the PIM electrolyte to maintain the electrochemical stability.^[^ ^22^ ^]^ The comparison between the gradual electrochemical floating analysis using NCM622||Li batteries in Figure 2d,e confirms that the highest withstand voltage for PIM electrolyte is 4.5 V, and when the voltage exceeds 4.5 V, the leakage current increases significantly. But the highest voltage for PEO is only 4.2 V. As known, owing to the instability in the PEO‐based SSE system, the interface between Li and PE will be worse under high voltage. At the same time, the higher cutoff voltage will deteriorate the crystalline structure of NCM cathode, which will make it collapsed and exhibit very fast capacity decay. Correspondingly, the Li ion transference number is also another index to observe the kinetics of Li ions. A significant increase of $t_{Li^{+}}$ is achieved from 0.26 for PEO and to 0.65 for PIM (Figure 2f and S4c,d, Supporting Information), almost three times higher than the controlled ones. Consequently, the enhanced kinetics of lithium ions are facilitated by the complexation of TFSI^−^ anions with the MOF's unsaturated [Zn_4_O] sites.^[10a]^ Meanwhile, the IL here acts as a wet bridge for Li‐ion transport, facilitating the movement of ions between the phases, and represents a potential pathway towards achieving high ionic conductivity.^[^ ^23^ ^]^

### 2.3 Fast Ion Transport and Electrochemical Stability for Long‐Term High‐Voltage Full Cell

**Figure 3:**
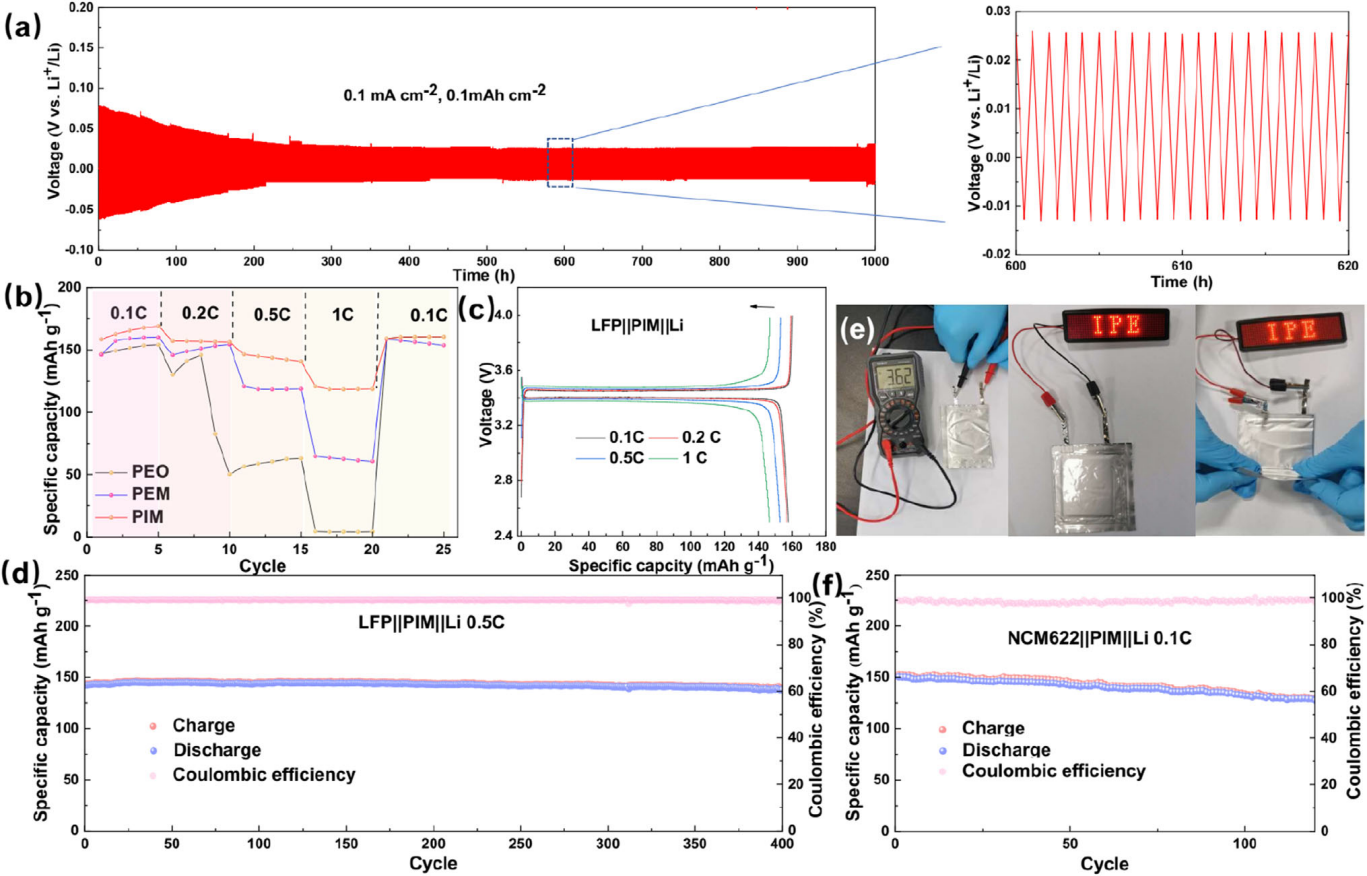
a) voltage profiles for the Li||PIM||Li symmetric battery at 0.1 mA cm^−2^. b) Rate performance of Li||PIM||LFP, Li||PEM||LFP, and Li||PEO||LFP cell. c) Galvanostatic charge/discharge curve of the Li||PIM||LFP cell at different rates. d) Cycle performance of the Li||PIM||LFP cell at 0.5 C. e) Pouch cell of LFP||PIM||Li battery with electrolyte PIM electrolyte at room temperature. f) Cycling performance of NCM622||PIM||Li cell at 0.1C with the upper voltage of 4.3V.

The interface stability of the PEO, PIM electrolytes, and Li metal anode was evaluated through Li plating/stripping tests using symmetric cells, separately. **Figure** **3**a shows that the Li||PIM||Li symmetric cell is capable of maintaining superior cycling stability for up to 1000 h at 0.1 mA cm^−2^ with an areal capacity of 0.1 mA h cm^−2^ under 60 °C. The overpotential was decreased gradually from 70 mV to around 38 mV after 200 h, indicating the smooth transfer of Li ion between the Li electrode and PIM. In contrast, the Li–Li symmetric cell with PEO membrane electrolyte can only survive for less than 250 h with a sudden voltage drop to less than 5 mV (Figure S5, Supporting Information), which can be considered a short circuit with the growth of dendritic Li. Enhancing the current density to 0.2 mA cm^−2^, the polarization voltage of the symmetric cell with PIM electrolyte kept around 60 mV and stabilized for 800 h (Figure S6, Supporting Information). After cycling, the electrochemical impedance spectroscopy (EIS) was obtained from the designate Li||PIM||Li cell before and after plating/stripping process. As exhibited in Figure S7, Supporting Information, the charge transfer resistance was slightly decreased from 86 to 75 Ω after cycling, validating the better interface contact after several stripping/plating behaviors. This suggests that the MOF and IL in PIM films could not only serve as a viable approach to attain uniform Li deposition but also bridge the excellent compatibility between the PIM electrolyte and Li metal. The contact angles between electrolyte slurry and the positive and negative electrodes can be indexed as shown in Figure S8, Supporting Information. With the addition of IL, the contact angles on both the positive and negative electrodes are significantly reduced, indicating that the IL@MOF nanostructure can improve the interface between the electrolyte and electrode materials, promoting multipoint deposition of lithium, effectively suppressing the formation of lithium dendrites.

Successively, the Li||LFP and Li||NCM622 full cells were assembled with 250 μm Li foil. All the cells are evaluated under the temperature of 60 °C. Figure 3b compared the rate performance of Li||PIM||LFP cells employed with PEO, PEM, and PIM electrolyte, respectively. All capacities are decreased slowly when increasing the current rates. The cell employed with PIM electrolyte displays the capacity of 168.9, 157.4, 146.6, and 121.5 mAh g^−1^ at 0.1, 0.2, 0.5, and 1 C, respectively (Figure 3c). However, at 1 C, PIM‐based battery exhibits the best performance than other two systems. Meanwhile, the optimized cell also keeps the stable plateau at various rate. As depicted in Figure 3d, the Li||PIM||LFP cell had a relatively stable specific discharge capacity from 142 to 138 mAh g^−1^ after 400 cycles with a high capacity retention of 97.2% at 0.5C. Figure S9, Supporting Information, also shows the stable galvanostatic voltage curve of the Li||PIM||LFP cell at 0.5 C. Decreasing the current rate, the Li||PIM||LFP cell displays the initial capacity of 151.9 mAhg^−1^ and keeps near 100% capacity retention at 0.2 C with the lasting lifespan for about 280 cycles (Figure S10, Supporting Information). The pouch cell of Li||PIM||LFP was also assembled and showcased in room temperature (Figure 3e, Supporting Information). As observed, the open circuit voltage of the pouch battery is 3.62 V and can power the series of LED lights after being charged. Moreover, the LED remains light even after the battery was bent, indicating the possibility of membrane electrolyte for flexible devices. In order to witness the electrolyte in high voltage, the Li||NCM622 cells with PIM electrolyte were assembled to evaluate their robustness. As illustrated in Figure 3f and S11, Supporting Information, the as‐prepared cell exhibited a discharge capacity of 150.4 mAh g^−1^ at a 0.1 C within the voltage range of 2.8–4.3 V at 60 °C. After 120 cycles, a discharge capacity of 127.5 mAh g^−1^ is remained, and the high capacity‐retention of 85.3% and high Coulombic efficiency are reserved, demonstrating the superior stability under high voltage of 4.3 V. The extended performance has demonstrated that the IL coupled with MOF acts as bridge between the electrode and polymer‐based electrolyte, which decreases the Li ion transport barrier. On the other hand, the IL also helps to react with metallic Li with the reduction of anion to form the uniform ion‐conductive SEI layer on Li metal surface.

### 2.4 Ex Situ Characterizations of Electrode/Electrolyte Interfaces

**Figure 4:**
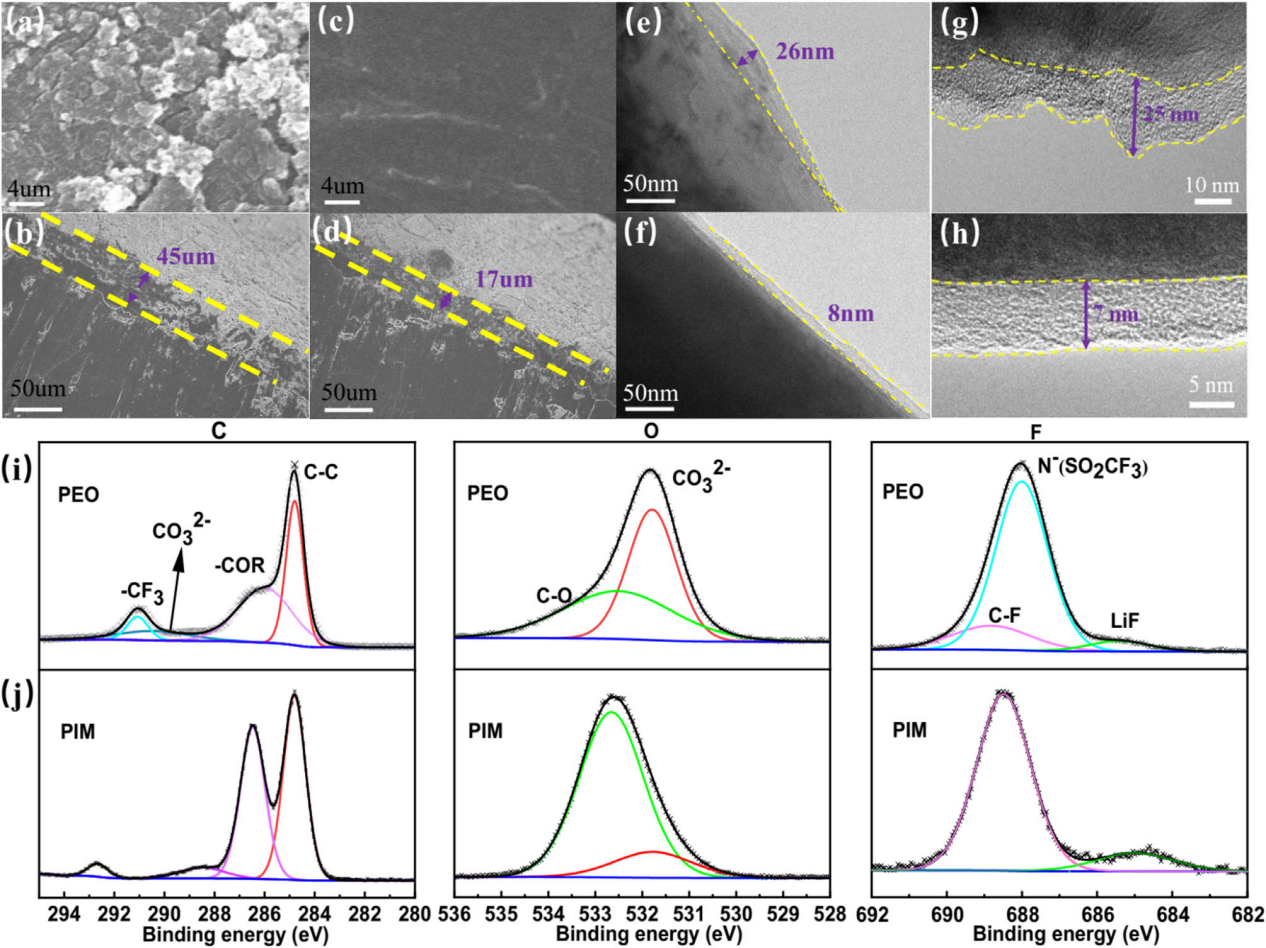
The a) top‐view and b) side‐view SEM images of lithium deposition based on PEO electrolyte. The c) top‐view and d) side‐view SEM images of lithium deposition based on PIM electrolyte. The TEM image of CEI layer on LFP cathode after 20 cycles based on e) PEO electrolyte and f) PIM electrolyte. The TEM image of CEI layer on NCM622 cathode after 20 cycles based on g) PEO electrolyte and h) PIM electrolyte. The high‐resolution of C1s, O1s, and F1s XPS spectra of LFP cathode based on i) PEO electrolyte and j) PIM electrolyte, respectively.

To probe the potential merits of PIM in full cell, the ex situ method of SEM, TEM, and XPS were carried out. The surfaces of lithium anode and cathode were observed after 20 cycles (**Figure** **4**). Employed with the PEO membrane electrolyte, large patches of moss‐like Li dendrites appeared on the surface and obvious cracks are exhibited, severely impacting the Li deposition behaviors with enhanced volumetric changes (Figure 4a,b). However, for the PIM membrane electrolyte system, the plating surface of Li anode was flat and smooth (Figure 4c) with a thickness of 17 μm (Figure 4d), proposing a uniform interface contact with solid‐state electrolyte. The absence of free anions adsorbed by MOFs enables Li ions to diffuse through the electrolyte membrane rapidly and uniformly before uniformly depositing on metallic Li surface. For the cathodic sides, the thickness of the formed cathode/electrolyte interphase (CEI) are recorded by TEM images, respectively. As depicted in Figure 4e, the CEI layer on LFP cathode in the PEO system is quite uneven with a maximum thickness of 26 nm, much worse than that of 8 nm for the PIM system (Figure 4f). However, under the high voltage cycling condition, the CEI in NCM622 cathode reflects good interfacial contact between the PIM electrolyte and the cathode, generating uniformly thin CEI layer about 7 nm in Figure 4 h. But it was up‐and‐down and increased to 25 nm in the PEO system (Figure 4 g), which was caused by poor interfacial compatibility.

In order to clearly make sense of the CEI layer, the XPS was used to detect the evolution in the CEI layers (Figure 4i,j). Both the C 1s and O1s spectra show that the signal assigned to CO_3_^2−^ around 298 eV and the peak at 531.8 eV was significantly decreased in Figure 4j in comparison with that in PEO‐based systems (Figure 4i). This intensity reduction proved that the formation of Li_2_CO_3_ component in CEI has been reduced in the PIM system owing to the sieving effect of MOFs. Further revealed in the F 1s spectrum, the peak located at 685 eV represents the formation of LiF, which is derived from the TFSI^−^ anions. The increased but uniform LiF with PIM electrolyte is beneficial for CEI to inhibit side reaction of PEO skeleton, thus showcasing the electrochemical stability.^[^ ^21^ ^]^

## 3 Conclusion

In summary, an interfacial Li^+^ diffusion booster in hybrid polymer electrolyte is achieved by wettable IL decorated MOFs. The TFSI^−^ anions are affected by positive metal atom centers and the PIM electrolyte exhibits exceptional high ionic conductivity up to 3.1 × 10^−3^ S cm^−1^ and high Li^+^ transference number up to 0.65. Consequently, the Li symmetric cells employed with PIM stabilize for over 1000 h with lower voltage hysteresis (25 mV) and the PIM electrolyte exhibits compatibility with the formation of stable LiF toward high‐voltage LiNi_0.6_Co_0.2_Mn_0.2_O_2_ and LiFePO_4_ electrodes, showing the capacity retentions of 85.3% and 97.2% after 120 and 400 cycles, respectively. This work suggests that the interfacial Li^+^ diffusion booster decorated polymer electrolyte has significant potential for enabling high‐voltage Li metal batteries.

## 4 Experimental Section

4.1

4.1.1

##### Materials

1‐Ethyl‐3‐methylimidazoliumbis(trifluoromethylsulfonyl)imide (EMIMTFSI, 993.99%, Lanzhou Institute of Chemical Physics, CAS), Zinc nitrate (Zn(NO_3_)_2_·6H_2_O, 99.9%, Aladdin), 1,4‐benzenedicarboxylate acid (H_2_BDC, 99%, Aladdin), anhydrous acetonitrile (AN, 99.6%, Fisher), N,N‐dimethylformamide (DMF, 99.9%, Aladdin), triethylamine (TEA, Aladdin), methylene chloride (CH_2_Cl_2_, 99.9%, Aladdin), polyethylene oxide (PEO, MW = 60 ≈ 106, sigma–Aldrich), lithium bis(trifluoromethanesulfonyl)imide (LiTFSI, 99.99%, Sigma–Aldrich), and lithium bis(fluorosulfonyl)imide (LiTFSI, 99.99%, Sigma–Aldrich).

##### Preparation of MOF‐5 and MOF‐IL

A solvothermal method was used to prepare nano‐sized MOF‐5 in this study.^[^ ^9^ ^]^ Specifically, 1.35 g Zn(NO_3_)_2_·6H_2_O and 0.25 g H_2_BDC were dissolved in 150 mL DMF in a 250 mL two‐neck flask. The mixture was treated with ultrasound and then the flask was equipped with a spherical condenser tube. The sealed apparatus was degassed using argon, and then 1 mL of TEA was injected into the reactor under argon atmosphere without stirring. The mixture was allowed to react for 7 h at 25 °C, resulting in a solution containing white precipitation. The precipitation was soaked in 100 mL DMF for 12 h with three times to remove unreacted Zn(NO_3_)_2_·6H_2_O and then soaked in 100 mL of CH_2_Cl_2_ for 12 h. The sample was dried in an evacuated oven at 150 °C for 12 h. Then, the MOF‐5 and IL EMIMTFSI were homogenized in a certain molar ratio by grinding in a mortar, followed by drying at 150 °C for 12 h to facilitate the infiltration of the IL into the pores of the MOF. The obtained IL@MOF‐5 nanoparticles were prepared.

##### Preparation of PEO‐MOF‐IL (PIM) Composite Solid Electrolytes

Fixed EO:Li^+^ at molar ratio of 16:1, PEO, LiTFSI, and different mass ratios of IL@MOF‐5 nanofillers were dissolved in AN solution and dispersed by ultrasound for 2 h. The contents of nanosized IL@MOF‐5 in PEO is 10 wt%. To create a homogenous emulsion, the suspension was continually swirled for 24 h. Then, resultant emulsion was applied on the PTFE plate. By vacuum drying at 50 °C for 12 h to remove the remaining solvent, the composite solid‐state electrolytes film with thickness of 80–100 micrometers was eventually produced. Also, PEO, and PEO‐IL@Mof‐5 (PEM) electrolytes were also prepared in the same way.

##### Material Characterizations

The FT‐IR spectra were obtained using a Nicolet Avatar 360 infrared spectrometer with a thermal detector, in the wavenumber range of 4000–500 cm^−1^. The surface morphology and cross‐sections of the samples were analyzed using SEM (Haitich SU8020, Japan). To examine the changes in the proportion of elements in the samples, an EDS was employed. The crystallinity of the samples was determined by using a Bruker D8 Focus X‐Ray diffractometer with Cu–Kα (λ = 0.15406 nm). To investigate the changes in the surface composition of cycled lithium metal, X‐ray photoelectron spectroscopy (XPS) was performed by using the Thermo Fisher Scientific ESCALAB 250 Xi instrument. AFM was used to assess the Young's moduli (Bruker Fastscabio). The Zeta potential of the MOF‐5 nanoparticle samples in acetonitrile solution was measured using a DelsaNano C instrument, and the ^1^H solid‐state NMR measurements were conducted on a JNM‐ECZ600R with a resonance frequency of 233 MHz.

##### Electrochemical Characterization

The samples were placed between two stainless steel disc electrodes, and the ionic conductivity was determined by measuring the electrochemical impedance spectroscopy (EIS) curves, including a frequency range of 0.01 Hz to 100 kHz, an amplitude of 10 mV, and an applied DC voltage of 0 V. The electrochemical stability window of the electrolyte was assessed at 60 °C using linear sweep voltammetry (LSV) with a scanning rate of 0.1 mV/s over a range of 2.0 V to 6.0 V. The ionic conductivity can be determined using the following equation: σ=L/(D*R), where R (Ω·cm^2^) represents the bulk resistance of the electrolyte, L represents the thickness of the electrolyte, and D represents the effective area. The transference number ($t_{Li^{+}}$) of the battery was determined via chronoamperometry using a constant polarization potential of 10 mV at 60 °C. The calculating equation of $t_{Li^{+}}$ is $t_{Li^{+}}=\left[I_{S}\left(\Delta V-R_{0}I_{0}\right)]/[I_{0}\left(\Delta V-R_{S}I_{S}\right)\right]$, where *I*_*o*_ and *I*_*s*_ correspond to the initial and steady‐state currents, *R*_*o*_ and *R*_*s*_ represent the interface resistances before and after polarization. A symmetrical lithium metal battery with a Li||PMI||Li configuration was assembled to investigate the compatibility of the electrolyte with a lithium metal anode at different current densities. Galvanostatic cycling tests were performed at a temperature of 60 °C. The as‐prepared LFP and NCM622 active cathode materials had a mass loading about 3 mg cm^−2^. The working voltage of LFP||PIM||Li batteries was from 2.5 to 4.0 V, and the NCM622||PIM||Li batteries was from 2.8 to 4.3 V. The voltage drift experiment was conducted by performing constant current and constant voltage tests on the NCM622||PEO||Li battery. The battery was charged with a constant current of 0.05mA to 4.0 V initially, then charged with 4.0 V for 10 h, and continuously charged with a constant current 0.05 mA to 4.1 V and held for 10 h. This process was repeated until the voltage reached to 5.0V. All the Li batteries were conducted on a Neware or LAND battery cycler.

## Conflict of Interest

The authors declare no conflict of interest.

## Supporting information

Supplementary Material

## Data Availability

The data that support the findings of this study are available from the corresponding author upon reasonable request.
