# Supplementary material for: Interfacial Li+ Diffusion Booster Accelerated by Enhanced Metal‐Organic Framework Sieving and Wettability for High‐Voltage Solid‐State Lithium Metal Batteries
Source: ChemSusChem. 2025 Sep 2;18(20):e202501351. doi: 10.1002/cssc.202501351 (PMC12548943; doi:10.1002/cssc.202501351)
Supplement: Supplementary file 1 — Supplementary Material [file CSSC-18-e202501351-s001.pdf]

Supporting Information

Interfacial Li^+ Diffusion Booster Accelerated by Enhanced MOF Sieving and Wettability for High-Voltage Solid-State Lithium Metal Batteries

Tianhua Chen[1,3], *Yongzheng Zhang*[4], *Simeng Wang*[3], *Jin Li*[3], *Hongzhen Lin*[2], *Dusan Losic*[1]*, *Shimou Chen*[5]*, & *Jian Wang*[2,6,7,]*

[1] School of Chemical Engineering and Advanced Materials, The University of Adelaide, Adelaide SA 5005, Australia

[2] *i*-Lab & CAS Key Laboratory of Nanophotonic Materials and Devices, Suzhou Institute of Nano-Tech and Nano-Bionics, Chinese Academy of Sciences, Suzhou 215123, China

[3] Institute of Process Engineering, Chinese Academy of Sciences, Beijing 100190, China

[4] State Key Laboratory of Chemical Engineering, East China University of Science and Technology, Shanghai 200237, China

[5] State Key Laboratory of Chemical Resource Engineering, Beijing University of Chemical Technology, Beijing 100029, China

[6] Helmholtz Institute Ulm (HIU), Ulm D89081, Germany

[7] Karlsruhe Institute of Technology (KIT), Karlsruhe D76021, Germany

Corresponding authors:

dusan.losic@adelaide.edu.au (D. Losic),

chensm@mail.buct.edu.cn (S. Chen)

jian.wang@kit.edu; wangjian2014@sinano.ac.cn (J. Wang)

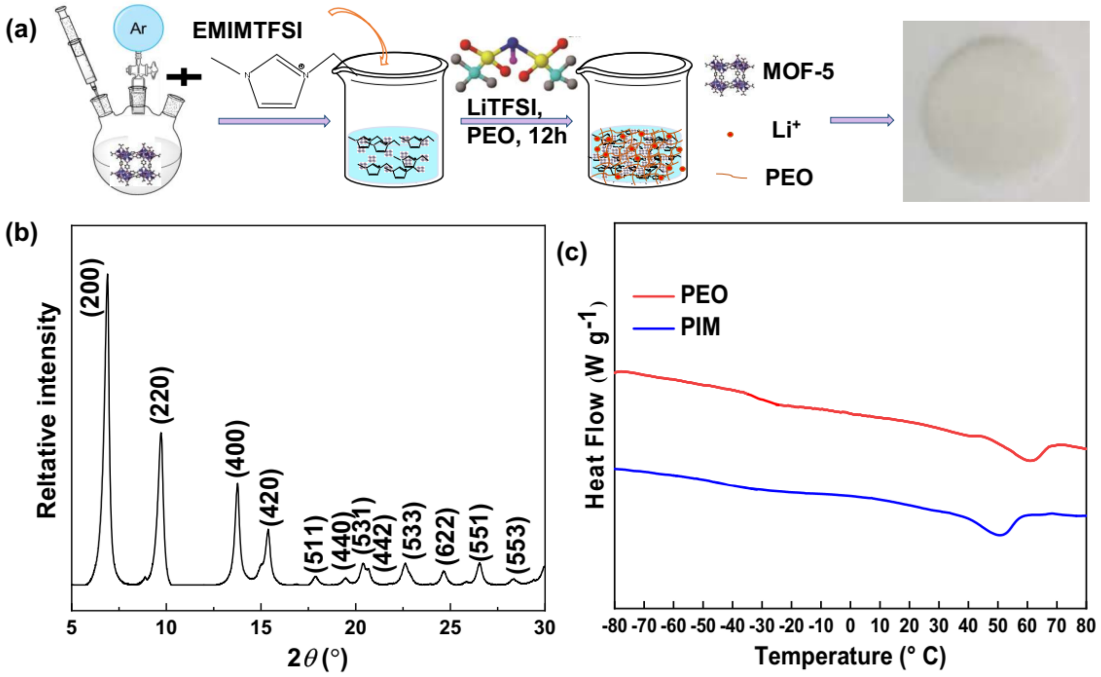

Figure S1. (a) Schematic fabrication illustration of PIM film, (b) XRD pattern of the MOF-5 sample, (c) DSC curve of PEO and PIM electrolyte.

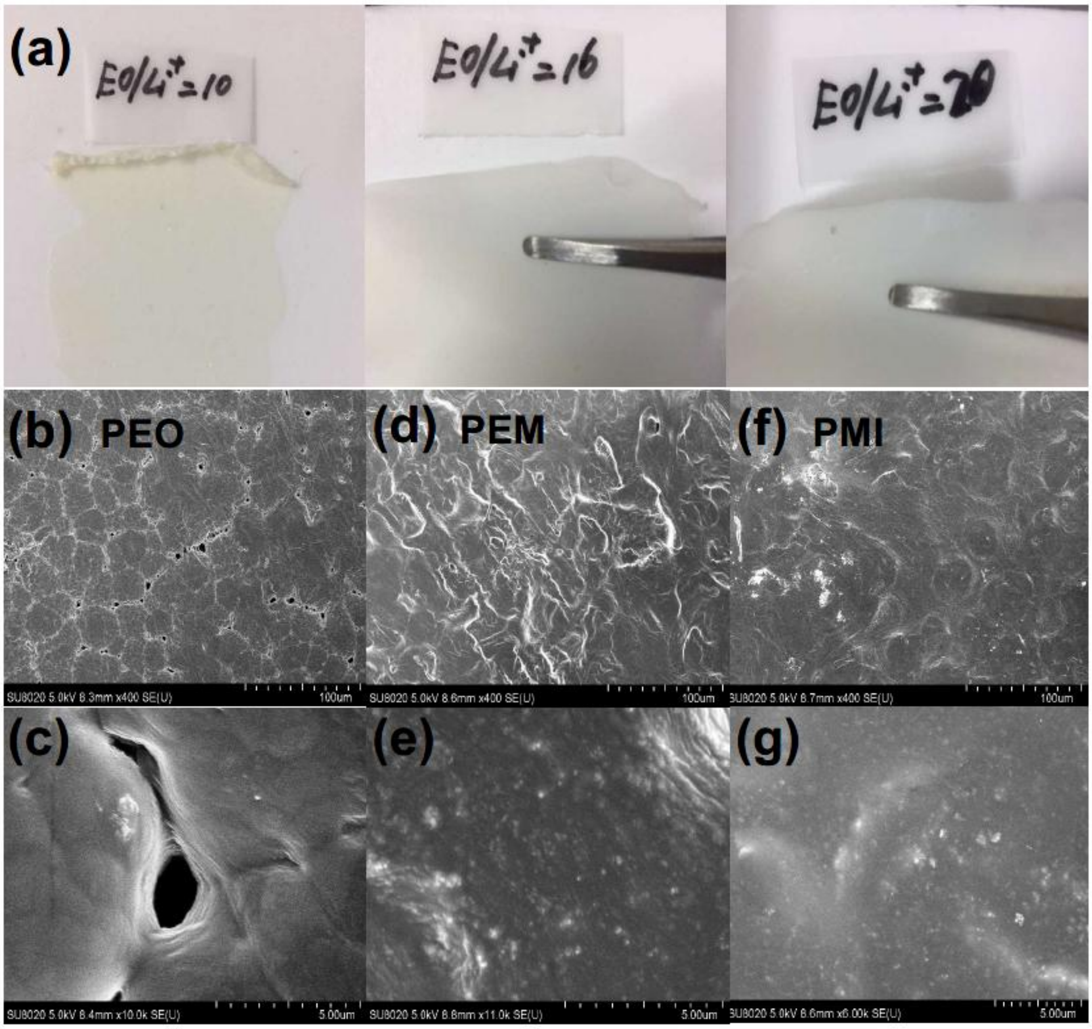

Figure S2. (a) Pictures of PIM films with different molar EO/Li^+ ratio SEM images of (b-c) PEO, (d-e) PEM, and (f-g) PIM electrolyte

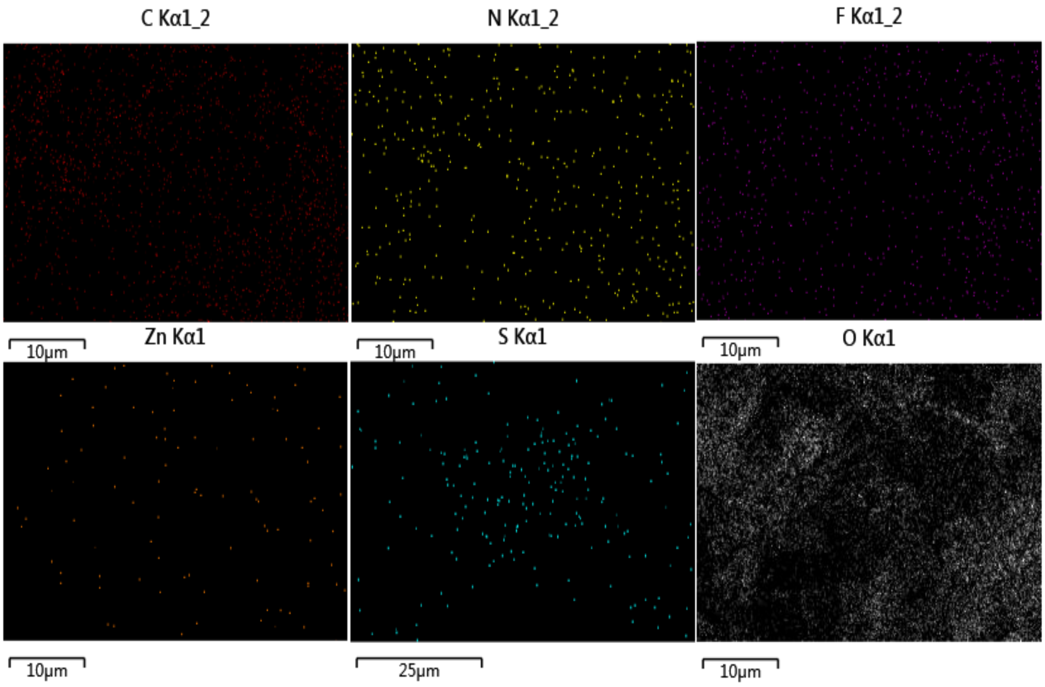

Figure S3. EDS mappings of distribution of different elements in the PIM membrane

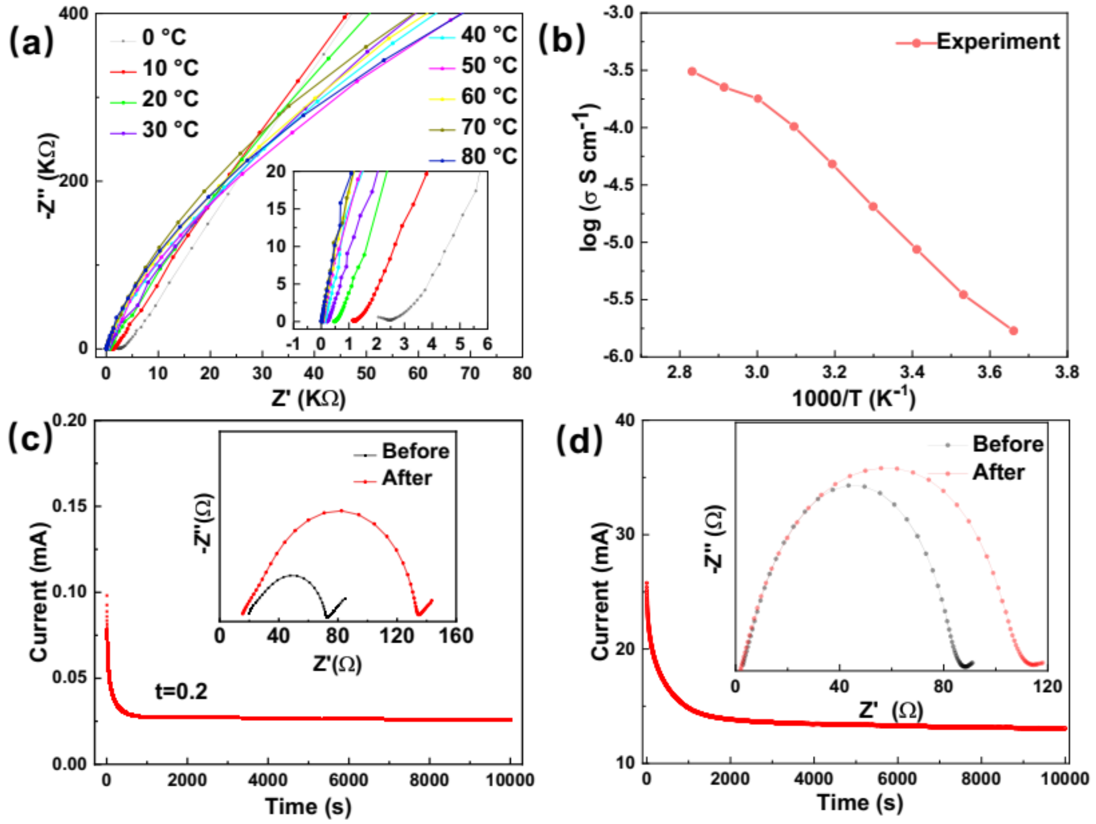

Figure S4. (a) electrochemical impedance spectroscopy plots of SS||PIM||SS batteries ranging from 0 - 80 °C, (b) corresponding Arrhenius plot of the ionic conductivity, Current-time curve following DC polarization curves of the (c) PEO electrolyte, (d) PIM electrolyte at 60 °C (inset: EIS variation at initial and steady states).

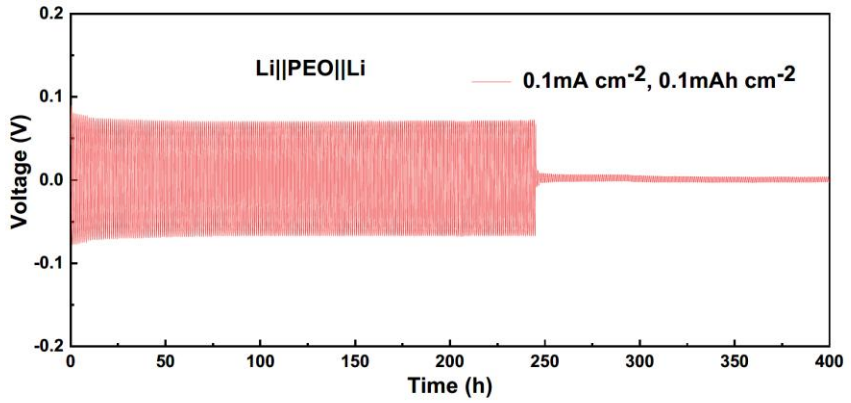

Figure S5. Voltage profiles for the Li||PEO||Li symmetric battery at current densities of 0.1 mA cm^{-2} at 60 °C.

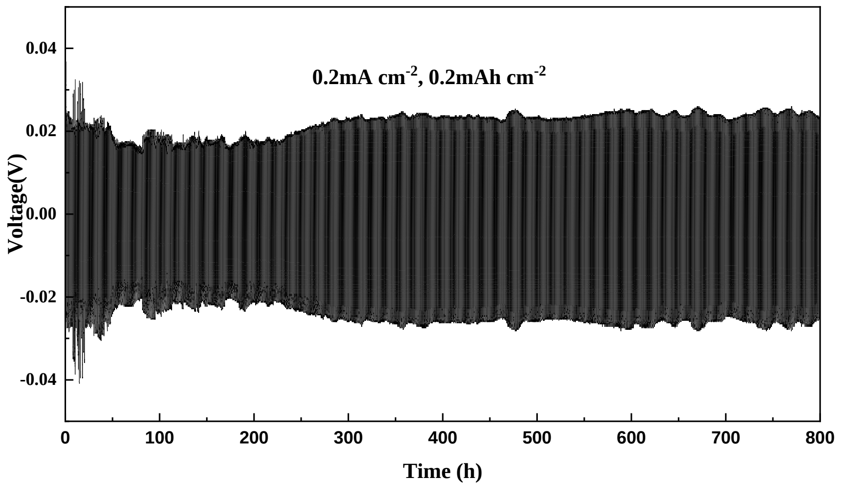

Figure S6. Voltage profiles for the Li||PIM||Li symmetric battery at current densities of 0.2 mA cm^{-2} at 60 °C.

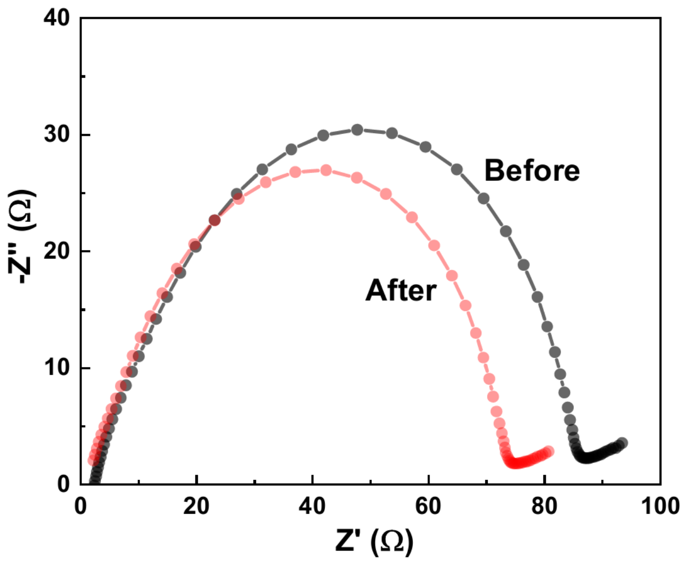

Figure S7. EIS before and after Li plating/stripping cycling for the Li||PIM||Li symmetric battery at current densities of 0.1 mA cm^{-2} at 60 °C.

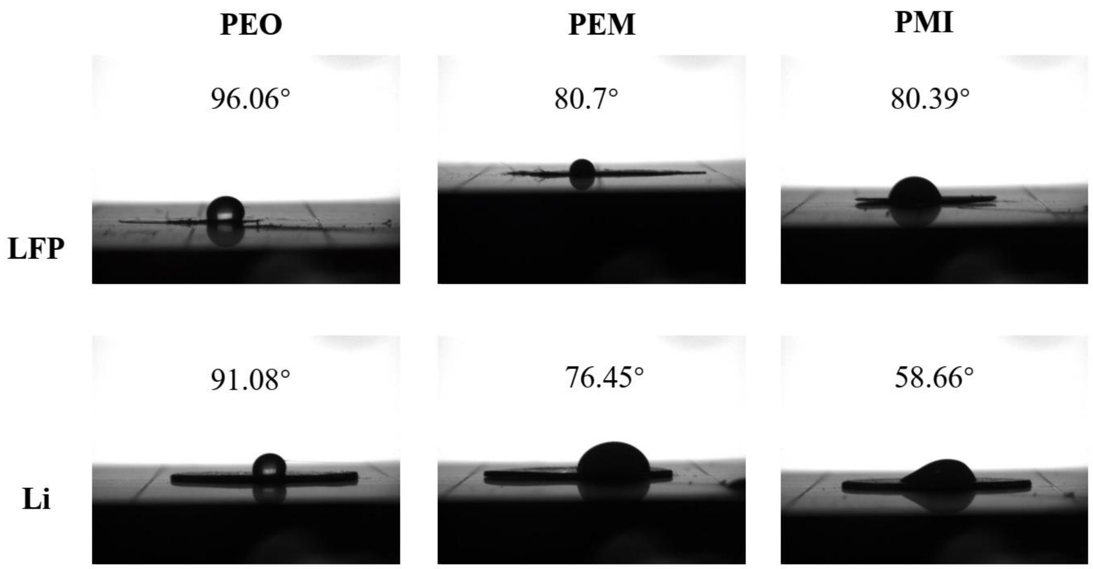

Figure S8. Contact angles of three electrolytes on the surface of LFP and Li anode

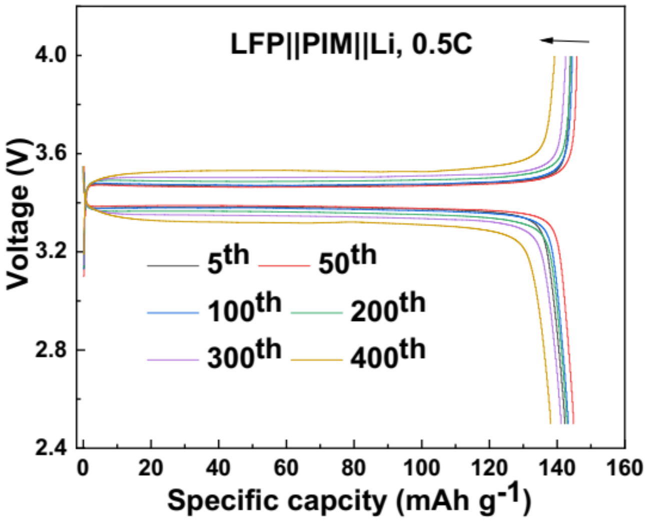

Figure S9. Galvanostatic charge/discharge curve of the Li||PIM||LFP cell at 0.5 C

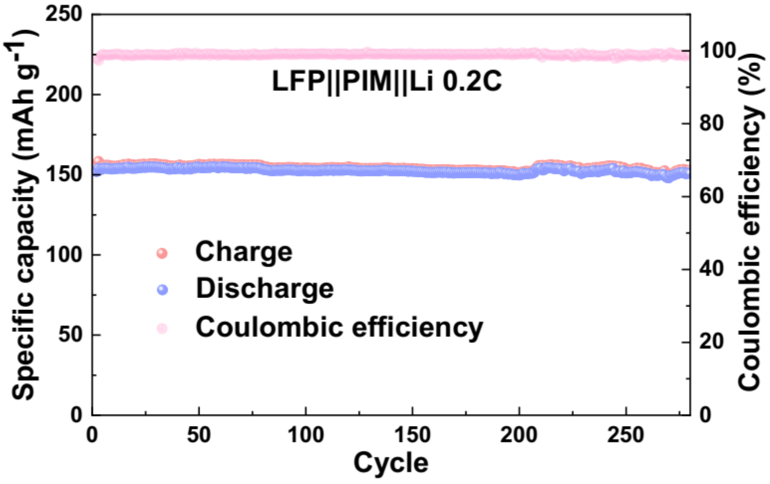

Figure S10. Galvanostatic charge/discharge curve of the Li||PIM||LFP cell at 0.2 C and 60 °C

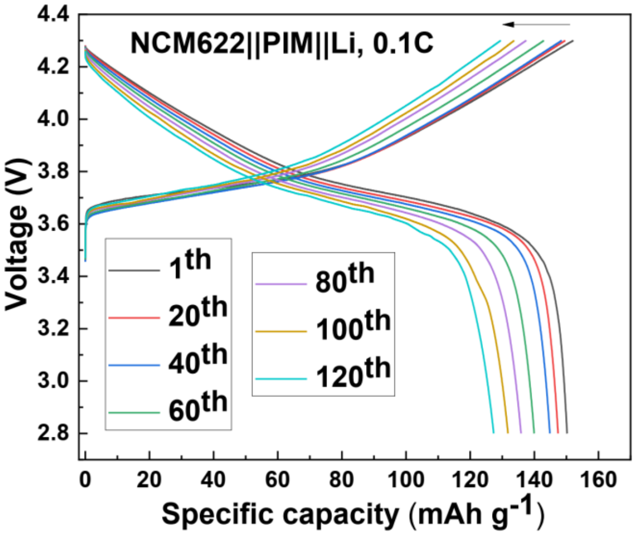

Figure S11. Galvanostatic charge/discharge curve of Li||PIM||NCM622 cell at 0.1 C
